# Memory and metamemory for social interactions: Evidence for a metamemory expectancy illusion

**DOI:** 10.3758/s13421-020-01071-z

**Published:** 2020-07-30

**Authors:** Laura Mieth, Marie Luisa Schaper, Beatrice G. Kuhlmann, Raoul Bell

**Affiliations:** 1 Department of Experimental Psychology, Heinrich Heine University Düsseldorf, Düsseldorf, Germany; 2 Department of Psychology, School of Social Sciences, University of Mannheim, Mannheim, Germany

**Keywords:** Cheater detection, Source memory, Metacognition, Judgments of item learning, Judgments of source learning

## Abstract

People do not always have accurate metacognitive awareness of the conditions that lead to good source memory. In Experiment 1, participants studied words referring to bathroom and kitchen items that were either paired with an expected or unexpected room as the source. Participants provided judgments of item and source learning after each item–source pair. In line with previous studies, participants incorrectly predicted their memory to be better for expected than for unexpected sources. Here, we show that this metamemory expectancy illusion generalizes to socially relevant stimuli. In Experiment 2, participants played a prisoner’s dilemma game with trustworthy-looking and untrustworthy-looking partners who either cooperated or cheated. After each round of the game, participants provided metamemory judgments about how well they were going to remember the partner’s face and behavior. On average, participants predicted their source memory to be better for behaviors that were expected based on the facial appearances of the partners. This stands in contrast to the established finding that veridical source memory is better for unexpected than expected information. Asking participants to provide metamemory judgments at encoding selectively enhanced source memory for the expected information. These results are consistent with how schematic expectations affect source memory and metamemory for nonsocial information, suggesting that both are governed by general rather than by domain-specific principles. Differences between experiments may be linked to the fact that people may have special beliefs about memory for social stimuli, such as the belief that cheaters are particularly memorable (Experiment 3).

When someone cheats on you, you may promise yourself to remember it forever. If that cheater has the facial appearance of a criminal, you may be particularly convinced that you will never forget what this person has done to you. Memory for another person’s behavior can be classified as *source memory* (i.e., memory for the context in which the person has been encountered; Johnson, Hashtroudi, & Lindsay, 1993). Metacognitive awareness of one’s own memory is crucial in deciding what to study and when to terminate study (for reviews, see Bjork, 1994; Bjork, Dunlosky, & Kornell, 2013; Soderstrom, Yue, & Bjork, 2016). Therefore, you may not put much effort into trying to memorize a person as a cheater if you expect your cheater memory to be particularly good. However, accurate memory predictions cannot be taken for granted. In fact, people’s metamemory—which includes their subjective beliefs and judgments about memory—is often dissociated from objective memory accuracy (e.g., Besken & Mulligan, 2013, 2014; Karpicke, Butler, & Roediger, 2009; Rhodes & Castel, 2008). Objective memory for cheating or cooperative behavior is particularly good when the behavior is unexpected (Bell & Buchner, 2012). Specifically, source memory is enhanced when a cheater has a trustworthy facial appearance (Bell, Buchner, Kroneisen, & Giang, 2012; Mieth, Bell, & Buchner, 2016). Such an *expectancy violation effect* on source memory has been found for nonsocial stimuli as well (Küppers & Bayen, 2014; Schaper, Kuhlmann, & Bayen, 2019a, 2019b). However, people are not metacognitively aware of this effect (Schaper et al., 2019a, 2019b). Instead, they are prone to a metamemory expectancy illusion as they predict memory for schematically expected information to be particularly good. The current research serves to (a) replicate the metamemory illusion for nonsocial stimuli, and (b) test whether the metamemory expectancy illusion generalizes to the social domain.

As yet, people’s metamemory beliefs about source memory have only been examined using nonsocial stimuli. Specifically, Schaper et al. (2019a, 2019b) showed participants words that referred to bathroom or kitchen items. The items were either presented with their schematically expected source (e.g., “oven in the kitchen”) or with an unexpected source (e.g., “frying pan in the bathroom”). Participants predicted better *item memory* for items presented with expected than with unexpected sources, but their veridical item memory was unaffected by schematic expectancy. Importantly, participants incorrectly predicted *source memory* to be better for expected than for unexpected item–source pairs. However, actual guessing-corrected source memory was better for unexpected than for expected sources. Schaper et al. (2019a, 2019b) attributed this metamemory expectancy illusion to global beliefs about the effects of relatedness on memory (Mueller, Tauber, & Dunlosky, 2013) and to an increased encoding fluency of the related item–source pairs (Undorf & Erdfelder, 2011, 2013, 2015). People hold the global belief that expected information is better remembered than is unexpected information (cf. Mueller et al., 2013; Schaper et al., 2019b). Furthermore, expected information is more fluently processed than unexpected information (Alter & Oppenheimer, 2009; Undorf & Erdfelder, 2015). Enhanced processing fluency at encoding in turn is associated with higher metamemory judgments. Ironically, veridical memory is often better for material that is more difficult to process because it requires more elaboration (e.g., Besken & Mulligan, 2013; Bjork, 1994; Rhodes & Castel, 2008; Yue, Castel, & Bjork, 2013). In source memory, people seem to fall prone to a metamemory illusion in that they systematically overestimate source memory for expected compared with unexpected sources (Schaper et al., 2019a, 2019b).

However, Schaper et al. (2019a, 2019b) used stimulus material of low social and emotional relevance. People thus may simply lack metamemory awareness in situations where accuracy is of little importance. Obviously, the ultimate goal of such research is not to specifically understand how people remember bathroom and kitchen items. Rather, we want to gain general insights into how expectations affect judgments of source learning and source memory. Although such generalizations are a necessary part of theorizing, they may represent dangerous simplifications. In a worst-case scenario, findings may fail to generalize beyond the specific type of material used in the original study.

Evolutionary psychology, in particular, criticizes mainstream psychology for postulating general-purpose mechanisms that can be applied to any kind of stimulus material independent of its specific type of content (e.g., Cosmides & Tooby, 1992). The present study serves to test whether the metamemory illusion can be obtained with stimulus material of higher evolutionary relevance. Specifically, we examined how well participants remembered the cheating or cooperative behavior of their partners in a prisoner’s dilemma game with real financial consequences. Successful reciprocal cooperation requires accurate memory for the cheating and cooperative behavior of others (Schaper, Mieth, & Bell, 2019). A great degree of precision in cheater detection is required: Cheating must not go unnoticed (Cosmides & Tooby, 1992). To achieve a high level of precision, people must be able to adaptively adjust their encoding resources to different situational demands (Kroneisen, Woehe, & Rausch, 2015). From an evolutionary perspective, metacognition is assumed to have evolved in humans because metacognitive reflections can be put to use in controlling how new information is encoded, what information is retrieved, how problems are solved, and how one interacts with other people (Metcalfe, 2008). Importantly, evolutionary psychology traditionally implies that important adaptive problems are dealt with by highly specialized cognitive modules that are specifically tailored to the requirements of these—narrowly defined—problems (Tooby & Cosmides, 2005). From these presumptions, the hypothesis can be derived that metacognition for social-exchange relevant information might be “special” in that it should be more accurate and less susceptible to illusions.

The present research provides an empirical test of whether the metamemory illusion generalizes to a paradigm with high social and emotional relevance. As a first step, we replicated the metamemory illusion using the same nonsocial material as Schaper et al. (2019a). In Experiment 1, bathroom items (e.g., a toothbrush) and kitchen items (e.g., an oven) were paired with the expected or unexpected source (“kitchen” or “bathroom”). Judgments of item learning (Rhodes, 2016) and judgments of source learning (Kuhlmann & Bayen, 2016) were assessed after each item–source pair had been encoded. In a following source monitoring test, we assessed how well participants recognized bathroom and kitchen items and how well they remembered the items’ sources. In Experiment 2, by contrast, trustworthy-looking and untrustworthy-looking faces were paired with cheating or cooperative behaviors in a sequential prisoner’s dilemma game with real financial consequences (cf. Bell et al., 2012; Mieth et al., 2016). This paradigm was structurally similar to that of Experiment 1 because the item information (e.g., a trustworthy-looking face) was paired with expected (e.g., cooperation) or unexpected (e.g., cheating) source information. Immediately after each round of the prisoner’s dilemma game, participants provided a judgment of item learning for the face and a judgment of source learning for the behavior. In a memory test, we assessed how well participants recognized the faces (item memory) and remembered the association between the faces and the cheating or cooperative behavior in the prisoner’s dilemma game (source memory).

We expected to replicate the metamemory illusion in source memory for nonsocial material (Schaper et al., 2019a, 2019b) in Experiment 1. The novel contribution of Experiment 2 is that we assessed metamemory judgments for item and source learning in the prisoner’s dilemma game. If metamemory judgments about social source memory are governed by the same principles as those about nonsocial source memory, people should show a metacognitive illusion. That is, they should predict source memory to be better for socially expected behaviors (e.g., a trustworthy-looking person cooperating) than for unexpected behaviors (e.g., a trustworthy-looking person cheating), due to the increased fluency associated with the processing of expected information and/or global beliefs that expected information is easier to remember (Schaper et al., 2019a, 2019b). Following Schaper et al. (2019a), we compared source memory between participants who provided the metamemory judgments and participants who did not provide any metamemory judgments during encoding, to test whether veridical memory is affected by the metamemory judgments. Schaper et al. (2019a) found the expectancy violation effect on source memory to be decreased when participants made metamemory predictions at encoding. Participants may elaborate on the relationship between the item and the expected source when making metamemory judgments, which selectively increases memory for expected information (Soderstrom, Clark, Halamish, & Bjork, 2015). If social source memory is equally affected by metacognitive processing as is nonsocial source memory, the source memory advantage for trustworthy-looking cheaters should decrease when metamemory judgments are provided. Following the procedure of Schaper et al. (2019a), we also assessed participants’ postdictions of their item and source memory after the memory test. These global judgments reflect the participants’ retrospective assessment of their memory for the different item–source combinations in the memory test. In Experiment 1, these postdictions should replicate the findings of Schaper et al. (2019a). In Experiment 2, they give us further insights about retrospective metamemory judgments concerning cheating and cooperative behaviors *after* the actual encoding episode and memory test. Experiment 3 complements Experiment 2 by providing insights about people’s beliefs about memory for cheaters and cooperators. Beliefs are global judgments about memory in the absence of first-hand experience with the specific stimulus material about which the judgments are made.

## Experiment 1

### Method

#### Participants

Up to nine participants were tested simultaneously in individual cubicles. We collected as much data as possible in the 4 weeks the laboratory was available to us. A total of 120 participants (75 female) were recruited on campus at Heinrich Heine University Düsseldorf. They were compensated with course credit or money. Upon arrival, participants were alternatingly assigned to one of two groups. The 60 participants (34 female) in the with-judgment group provided metamemory judgments at encoding, while the 60 participants (41 female) in the without-judgment group did not. Age ranged between 18 and 39 years (*M*_age_ = 24 years, *SD*_age_ = 4 years). All participants gave written informed consent in accordance with the Declaration of Helsinki.

A sensitivity power analysis calculated with G*Power (Faul, Erdfelder, Lang, & Buchner, 2007) showed that with a sample size of 120 participants (60 per group), 96 answers per participant in the memory test, and given α = .05, effect sizes of *w* = 0.05 (small effects) could be detected in the goodness-of-fit test of the model with a statistical power of 1 − β = .95.

### Materials

Materials were identical to those used by Schaper et al. (2019a). Items were highly expected for one room and highly unexpected for the other room. The 96 items were split into three lists, each consisting of 16 bathroom and 16 kitchen items. During encoding, items from one list were presented with the kitchen source, and items from the other list were presented with the bathroom source so that 32 items were paired with the expected source and 32 items were paired with the unexpected source. The items of the third list were used as distractors during the memory test. Across participants, all three lists were presented equally often with the kitchen, with the bathroom, and as distractors.

#### Encoding phase

The procedure (including the counterbalancing scheme) was largely identical to that used by Schaper et al. (2019a). However, a few adjustments were made to make the experiment more similar to the experiment using social stimulus material (Experiment 2). Prior to encoding, the memory test was not mentioned in the written instructions (other than in Schaper et al., 2019a). The encoding phase started with two buffer items (one in the kitchen, one in the bathroom) which were equally expected for both rooms. These buffer trials served to familiarize the participants with the procedure. Then the 64 items were presented in randomized order. Each trial started with the presentation of an item at the center of the screen. The item (without source information) was presented in white letters in standard German capitalization on a black background. In Schaper et al. (2019a), items and source information had been presented simultaneously. In the present experiment, by contrast, source information was shown only after a button press to make the procedure more similar to that of Experiment 2 reported here. Directly below the item, a button with the label “Where?” was presented. Upon clicking this button, participants were informed about whether this item was found “in the KITCHEN” or “in the BATHROOM” (rooms in capital letters).

#### Judgments of item and source learning

Metamemory judgments were assessed as in Schaper et al. (2019a). Participants in the with-judgments group were first asked to predict the probability that they would later remember the item (judgment of item learning). Specifically, they were asked, “How likely is it that you will later remember this item?” After that, a different screen appeared, and participants were asked to predict the probability that they would later remember the room in which the item was placed (judgment of source learning). Specifically, they were asked, “Provided that you remember this item, how likely is it that you will remember the room (kitchen or bathroom) in which this item was placed?” Each question was displayed in the middle of the screen, with items and sources no longer visible. Judgments were assessed on a continuous scale from 0% (“I will definitely *not* remember this”) to 100% (“I will definitely remember this”). Judgments were typed into a text field using the number pad of the keyboard. Participants were allowed to correct their responses. To make the judgments more discriminable, a different background color was used for the judgment of item learning and the judgment of source learning (blue or yellow, randomized between participants). Participants in the without-judgments group did not provide these judgments.

#### Source monitoring test

After encoding, participants received instructions for the memory test. A standard source monitoring test was applied that was identical to that used in previous studies (Schaper et al., 2019a, 2019b). The 64 items from the encoding phase were randomly intermixed with the 32 new (16 bathroom, 16 kitchen) items. The items were presented, one at a time, in random order at the center of the screen. For each item, participants indicated, by clicking on one of two response buttons, whether or not they had seen the item during encoding. If so, they further indicated whether the item had been presented in the kitchen or in the bathroom. Then the next trial started.

#### Measuring source memory

**Fig. 1:**
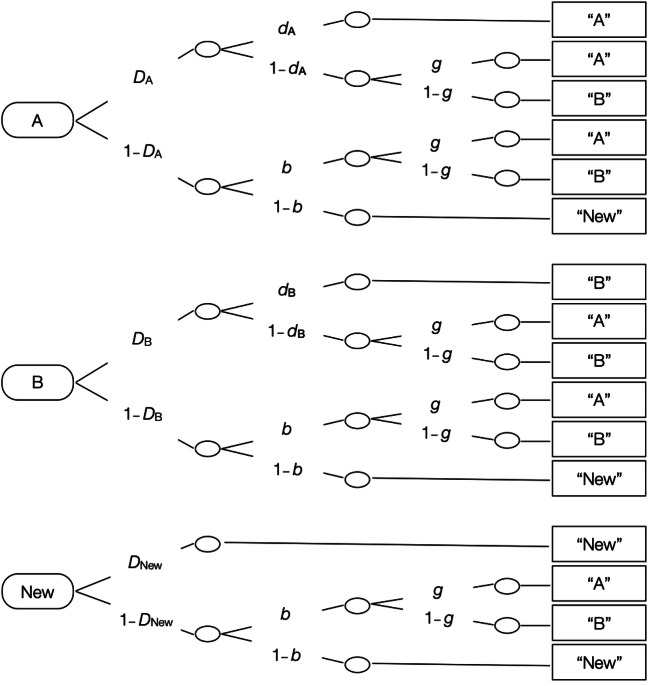
Multinomial model of source memory, adapted from Bayen et al. (1996). Rounded rectangles on the left (A, B, and New) represent the items that were shown in the memory test (items that were placed in the kitchen, items that were placed in the bathroom, and new items in Experiment 1, and cheater, cooperator, or new faces in Experiment 2). The letters along the branches represent the probabilities with which certain memory states occur (*D*: probability of recognizing an old item as old and a new item as new; *d*: conditional probability of remembering whether an old item was associated with Source A or Source B; *g*: conditional probability of guessing that the item was associated with Source A rather than with Source B; *b*: conditional probability of guessing that an item was old rather than new). Rectangles on the right reflect the participants’ answers in the memory test

When measuring source memory, it is important to disentangle source memory, item memory, and guessing processes (Bröder & Meiser, 2007). To this end, we used the multinomial source monitoring model (Bayen, Murnane, & Erdfelder, 1996). The model in Fig. 1 has been successfully validated and applied in many previous studies (Bayen & Kuhlmann, 2011; Bell et al., 2012; Kroneisen et al., 2015; Kuhlmann, Vaterrodt, & Bayen, 2012; Küppers & Bayen, 2014). The parameters are probabilities that vary between zero and one. The three trees of the model represent the different types of items presented in the memory test. Participants saw old items that had either been presented in the kitchen or in the bathroom and new items. The first tree represents the processes that occur in response to Source A items in the memory test. In the present experiment, Source A stands for the kitchen source, and Source B stands for the bathroom source. With probability *D*_A_, participants recognize the item as old. With the conditional probability *d*_A_, participants also have source memory (and thus remember that the item was presented in the kitchen). When participants have no source memory (with probability 1 − *d*_A_), they have to guess with probability *g* that the item was presented in Source A (the kitchen) or with the complementary probability 1 − *g* that the item was presented in Source B (in the bathroom). When participants do not recognize the item with probability 1 − *D*_A_, they guess with probability *b* that the item is old and have to guess with probability *g* that the item was presented in Source A and with 1 − *g* that the item was presented in Source B. Alternatively, participants guess that the item is new with probability 1 − *b*. Response frequencies from the memory test are used to estimate the parameters for item memory (*D*), item guessing (*b*), source memory (*d*), and source guessing (*g*). For the current study, four sets of model trees were needed for each combination of item type (bathroom items and kitchen items) and judgment group (without judgments and with judgments).

#### Postdictions

After the memory test, participants provided postdictions (Schaper et al., 2019a), which are aggregated judgments of item memory and source memory. Postdictions were assessed for all four item type (bathroom items and kitchen items) × source type (in the kitchen and in the bathroom) combinations. The order of the postdictions was randomized with the restriction that the item-memory postdictions for a given item type × source type combination (e.g., “How likely was it that you have remembered the kitchen-typical items that were placed in the bathroom? [Remembering here only means that you have remembered having seen the item; it does not matter whether you also have correctly remembered the room in which it was placed.]”) were always immediately followed by the corresponding source-memory postdictions for that combination (e.g., “Staying with kitchen-typical items that were placed in the bathroom: For what percentage of those items that you have recognized did you correctly remember that they were placed in the bathroom?”). The rating scale ranged from 0% (“I have definitely not remembered this”) to 100% (“I have definitely remembered this”). Answers were typed into a text field using the number pad of the keyboard. Participants were allowed to correct their responses.

#### Design

We used a 2 × 2 × 2 design with item type (bathroom items vs. kitchen items) and source type (in the kitchen vs. in the bathroom) as within-subject factors and judgment group (without-judgments vs. with-judgments) as between-subjects factor. Dependent variables were metamemory judgments (judgments of item and source learning) averaged across items, objective memory (item and source memory), guessing, and postdictions (of item and source memory).

### Results

Metamemory judgments were analyzed with repeated-measures analyses of variance (ANOVAs). Source monitoring processes were analyzed with the multinomial model shown in Fig. 1. Parameter estimates and goodness-of-fit tests were calculated with multiTree (Moshagen, 2010).^1^ All analyses use significance levels of .05, except for the supplementary analyses following up on the significant interactions, which use a Bonferroni-corrected significance level of .025.

#### Judgments of item and source learning

**Fig. 2:**
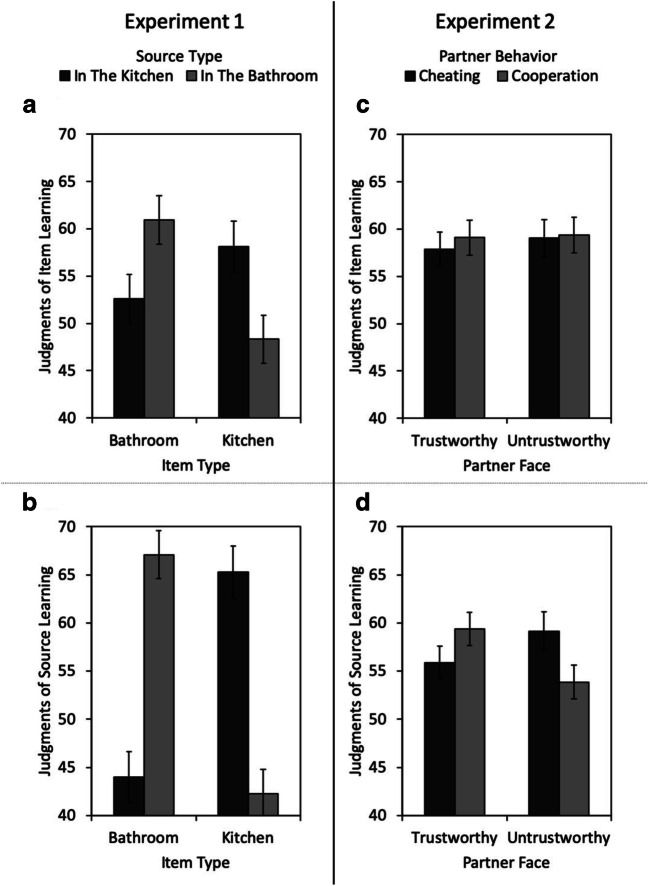
Judgments of item learning (**a, c**) and judgments of source learning (**b, d**) as a function of the item type (bathroom items vs. kitchen items) and source type (in the kitchen vs. in the bathroom) in Experiment 1 (left panels) or as a function of the partner’s facial trustworthiness (trustworthy vs. untrustworthy) and the partner’s behavior (cheating vs. cooperation) in Experiment 2 (right panels). Error bars represent the standard errors of the means

In the with-judgments group, judgments of item learning were influenced by item type, *F*(1, 59) = 17.71, *p* < .001, η_p_^2^= 0.23, but not by source type, *F*(1, 59) = 0.97, *p* = .330, η_p_^2^= 0.02. There was an interaction between item type and source type, *F*(1, 59) = 26.57, *p <* .001, η_p_^2^= 0.31 (see Fig. 2a). On average, participants predicted better item memory for bathroom items presented in the bathroom than for bathroom items in the kitchen, *F*(1, 59) = 17.88, *p <* .001, η_p_^2^= 0.23, and for kitchen items in the kitchen than for kitchen items in the bathroom, *F*(1, 59) = 28.98, *p <* .001, η_p_^2^= 0.33.

Judgments of source learning were also influenced by item type, *F*(1, 59) = 5.64, *p* = .021, η_p_^2^= 0.09, but not by source type, *F*(1, 59) < 0.01, *p* = .962, η_p_^2^< 0.01. Critically, there was an interaction between item type and source type, *F*(1, 59) = 68.30, *p* < .001, η_p_^2^= 0.54. On average, participants predicted better source memory for bathroom items in the bathroom than for bathroom items in the kitchen, *F*(1, 59) = 62.09, *p* < .001, η_p_^2^= 0.51, and for kitchen items in the kitchen than for kitchen items in the bathroom, *F*(1, 59) = 66.47, *p* < .001, η_p_^2^= 0.53 (see Fig. 2b). Thus, participants predicted better item and source memory for expected pairings than for unexpected pairings.

#### Item and source memory

**Fig. 3:**
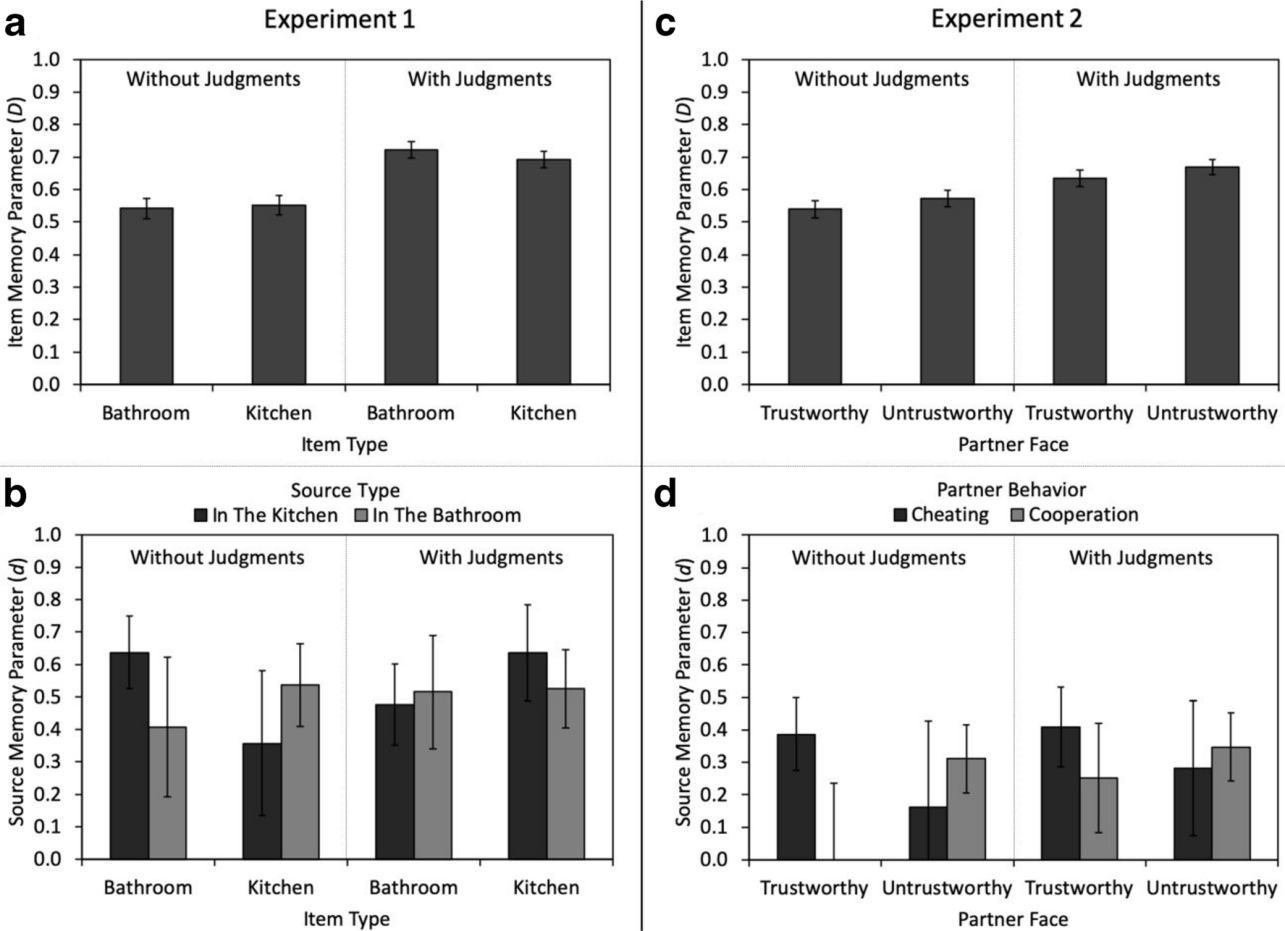
Parameter estimates for the item memory parameter *D* (**a, c**) and source memory parameter *d* (**b, d**) as a function of the item type (bathroom vs. kitchen) and source type (in the kitchen vs. in the bathroom) in Experiment 1 (left panels) and as a function of the partners’ facial trustworthiness (trustworthy vs. untrustworthy) and the partners’ behavior (cheating vs. cooperation) in Experiment 2 (right panels). The error bars represent 95% confidence intervals

Two-high threshold models (Bayen et al., 1996; Snodgrass & Corwin, 1988) commonly imply that item memory does not differ between old and new items to obtain identifiability. Therefore, we incorporated the restriction *D*_A_ = *D*_B_ = *D*_New_ into the model displayed in Fig. 1. This restriction also implies that item memory does not differ between items presented in the kitchen and items presented in the bathroom, which was supported by a repeated-measures ANOVA performed on the corrected hit rate for item memory (given by hit rate minus false alarm rate; Snodgrass & Corwin, 1988) of the present experiment. The base model including these restrictions was compatible with the data, *G*^2^(4) = 3.71, *p* = .446. There was no difference in item memory between bathroom and kitchen items, Δ*G*^2^(2) = 2.80 *p* = .247, *w* = 0.02 (see Fig. 3a). However, participants in the with-judgments group showed better item memory than participants in the without-judgments group, Δ*G*^2^(2) = 124.37 *p* < .001, *w* = 0.10. Consistent with previous studies (Rhodes, 2016; Schaper et al., 2019a), the requirement to invest processing resources and time into the metacognitive judgments benefitted item memory.

Source-memory parameter *d* represents the conditional probability that the room in which an item was placed was successfully remembered, provided that the item had been recognized as old. Following the procedure of Schaper et al. (2019a), we started the analysis by equating the two parameters representing source memory for expected item–source pairings, as well as the two parameters representing source memory for unexpected item source pairings. The resulting model provided a satisfactory fit to the data, *G*^2^(8) = 11.68, *p* = .166, so that it was used as the new base model for the subsequent comparisons of the source memory parameters (see Erdfelder et al., 2009). As in the study of Schaper et al. (2019a), participants in the without-judgments group showed an expectancy violation effect. Their source memory was better for unexpected item–source pairings than for expected item–source pairings, Δ*G*^2^(1) = 3.96, *p* = .047, *w* = 0.02 (left side of Fig. 3b). By contrast, participants in the with-judgment group did not show such an expectancy violation effect. Their source memory did not differ between unexpected and expected item–source pairings, Δ*G*^2^(1) = 0.49, *p* = .483, *w* < 0.01 (right side of Fig. 3b). However, a direct comparison of the parameters reflecting source memory of expected and unexpected pairs across groups did not support that source memory differed significantly between groups, Δ*G*^2^(2) = 3.96, *p* = .138, *w* = 0.02. At a descriptive level, these findings are consistent with those of previous studies showing that metamemory judgments selectively enhance memory for expected information (Schaper et al., 2019a; Soderstrom et al., 2015), but this difference was not substantiated by the model-based test.

Source guessing parameters are reported in Table 1. When participants did not remember in which room an item was placed, they had to guess whether it was placed in the kitchen or in the bathroom. Parameter *g* (reflecting the probability of guessing that an item was placed in the kitchen) differed significantly between bathroom and kitchen items, independent of whether participants provided metamemory judgments, Δ*G*^2^ (1) = 8.02, *p* = .005, *w* = 0.03, or not, Δ*G*^2^(1) = 14.36, *p* < .001, *w* = 0.04. This provides evidence of a schematic guessing bias in both groups.

**Table 1:** Guessing parameter estimates (and 95% confidence intervals) as a function of the item types (bathroom vs. kitchen) and judgment group (without judgments vs. with judgments) in Experiment 1

Without judgments		With judgments	
Bathroom	Kitchen	Bathroom	Kitchen
Parameter *b*			
0.37 (0.34–0.41)	0.26 (0.22–0.29)	0.36 (0.31–0.41)	0.32 (0.28–0.37)
Parameter *g*			
0.37 (0.30–0.45)	0.60 (0.51–0.69)	0.40 (0.30–0.49)	0.60 (0.50–0.70)

*Note.* Parameter *b* represents the probability of guessing that an item was old. Parameter *g* represents the probability of guessing that an item was placed in the kitchen rather than in the bathroom

#### Postdictions of item and source memory

**Fig. 4:**
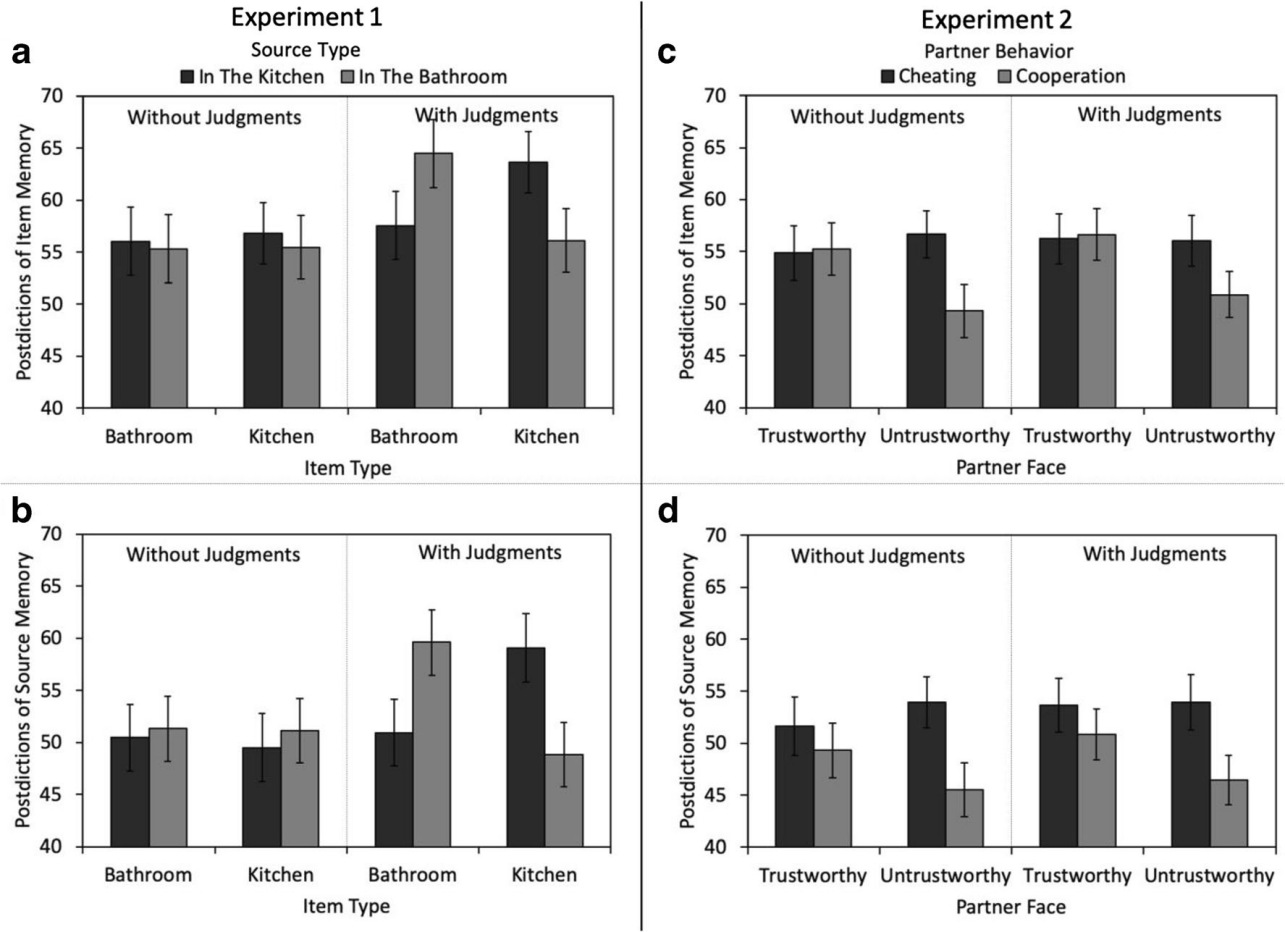
Postdictions of item memory (**a, c**) and postdictions of source memory (**b, d**) as a function of the item type (bathroom vs. kitchen) and source type (in the kitchen vs. in the bathroom) in Experiment 1 (left panels) or as a function of the partner’s facial trustworthiness (trustworthy vs. untrustworthy) and partner’s behavior (cheating vs. cooperation) in Experiment 2 (right panels). The error bars represent the standard errors of the means

After the memory test, participants in both groups provided postdictions (aggregated judgments about item and source memory) for all four combinations of the design. Data of 18 participants were incomplete and had to be excluded from this analysis. Postdictions for item and source memory are reported in Figs. 4a–b. In the analysis of the postdictions of item memory, there was neither a main effect of item type nor a main effect of source type. Both variables did not significantly interact with judgment group (all *F*s ≤ 0.48). The interaction between item type and source type was significant. Participants thought they had remembered items with expected sources better than items with unexpected sources, *F*(1, 100) = 6.08, *p* = .015, η_p_^2^= 0.06. However, a three-way interaction, *F*(1, 100) = 5.14, *p* = .026, η_p_^2^= 0.05, reflected the fact that this expectancy illusion was only present in the with-judgments group, *F*(1, 50) = 9.73, *p* = .003, η_p_^2^= 0.16, but not in the without-judgments group, *F*(1, 50) = 0.02, *p* = .880, η_p_^2^< 0.01.

Similar results were obtained for the postdictions of source memory. There was neither a main effect of item type nor a main effect of source type. Both variables did not significantly interact with judgment group (all *F*s ≤ 0.81). There was an interaction between item type and source type. Participants thought they had remembered expected sources better than unexpected sources, *F*(1, 100) = 6.62, *p* = .012, η_p_^2^= 0.06. A three-way interaction, *F*(1, 100) = 7.78, *p* = .006, η_p_^2^= 0.07, reflected that this expectancy illusion was only present in the with-judgments group, *F*(1, 50) = 11.61, *p* = .001, η_p_^2^= 0.19, but not in the without-judgments group, *F*(1, 50) = 0.03, *p* = .861, η_p_^2^< 0.01.

### Discussion

Despite minor changes in the procedure (i.e., presenting the item first and then the source), we successfully replicated the metamemory expectancy illusion—first discovered by Schaper et al. (2019a)—in judgments of item and source learning in Experiment 1. Participants predicted on average better memory for both items and sources when the item was presented with the expected source than when the item was presented with the unexpected source. In stark contrast to these metamemory predictions, source memory was actually better for unexpected than for expected item–source pairings in the without-judgment group. The source memory advantage for unexpected information was only present in the without-judgments group and absent in the with-judgments group. Even though the direct comparison between groups was not significant, the pattern of results is consistent with the findings of Schaper et al. (2019a) at a descriptive level. In the with-judgments group, the metamemory expectancy illusion persisted in the postdictions that were assessed after the memory test. The without-judgments group showed no such effect. This suggests that providing metamemory judgments during encoding increased the conviction that expected information improved memory. All of these results replicate those of Schaper et al. (2019a). Having established the robustness of these findings, the next aim is to test whether the metamemory illusion generalizes from the nonsocial domain to the social domain.

## Experiment 2

To test whether the metamemory illusion observed in Experiment 1 generalizes to a different stimulus domain, we used a sequential prisoner’s dilemma game (Clark & Sefton, 2001) to pair trustworthy-looking and untrustworthy-looking faces with cheating and cooperative behavior in Experiment 2. As in Experiment 1, the item information (e.g., a trustworthy face) was associated with either unexpected or expected source information (cheating or cooperative behavior). As in Experiment 1, we assessed participants metamemory in judgments of item learning (Rhodes, 2016) and judgments of source learning (Kuhlmann & Bayen, 2016) after each round of the prisoner’s dilemma game. In a subsequent memory test, we assessed how well participants remembered the partners’ faces (item memory) and the associated cheating or cooperative behavior (source memory). After the memory test, participants were asked to provide postdictions for item and source memory in all four cells of the design.

### Method

#### Participants

We collected as much data as possible in the 6 weeks the laboratory was available to us. A total of 185 participants (124 female) took part in Experiment 2. They were compensated with course credit or money. They were alternatingly assigned to either the with-judgment group (*n* = 92, 59 female) or without-judgment group (*n* = 93, 65 female). Three additional data files had to be excluded because of repeated participation. Age ranged between 17 and 40 years (*M*_age_ = 23 years, *SD*_age_ = 4 years). All participants gave written informed consent in accordance with the Declaration of Helsinki.

A sensitivity power analysis showed that with a sample size of 185 participants (>90 per group), 80 answers per participant in the memory test, and given α = .05, effect sizes of *w* = 0.04 could be detected in the model-based comparisons with a statistical power of 1 − β = .95. This power analysis was calculated using G*Power (Faul et al., 2007).

#### Prisoner’s dilemma game

Participants played a one-shot sequential prisoner’s dilemma game with 20 trustworthy-looking and 20 untrustworthy-looking partners. For each participant, the faces of the partners were drawn from a pool of 80 frontal facial photographs of women with neutral facial expressions (250 × 375 pixels) from the FERET database (Phillips, Wechsler, Huang, & Rauss, 1998). Of these faces, 40 were trustworthy looking and 40 were untrustworthy looking according to an independent norming study with *N* = 21 students. The faces were identical to those used in a previous study in which participants showed enhanced source memory for trustworthy-looking cheaters (Mieth et al., 2016). Mean trustworthiness ratings in the norming study on a scale ranging from 1 to 6 were *M* = 4.28 (*SD* = 0.23; min = 4.00; max = 4.86) for the trustworthy-looking and *M* = 2.75, (*SD* = 0.24; min = 1.90; max = 3.10) for the untrustworthy-looking faces. The faces were randomly drawn from the pool of 80 faces and randomly assigned to the conditions with the restriction that half of the cheating partners and half of the cooperating partners looked trustworthy, while the other half looked untrustworthy.

The prisoner’s dilemma game was identical to that used in previous studies (Bell et al., 2012; Bell, Giang, Mund, & Buchner, 2013; Mieth et al., 2016). Participants were required to invest money into a joint business venture with partners whose faces were shown on the screen. Participants knew that they played for real money (relative to an initial endowment of 100 cents, they could win or lose money depending on their own and their partners’ decisions). In each trial, participants were required to invest a small amount of money (30 or 15 cents). Cooperating partners always reciprocated the participants’ investment, which led to a small gain for both partners (10 or 5 cents). Cheating partners invested nothing which led to a comparatively large gain for the partner (20 or 10 cents) at the expense of the participant who lost money (−10 or −5 cents).

**Fig. 5:**
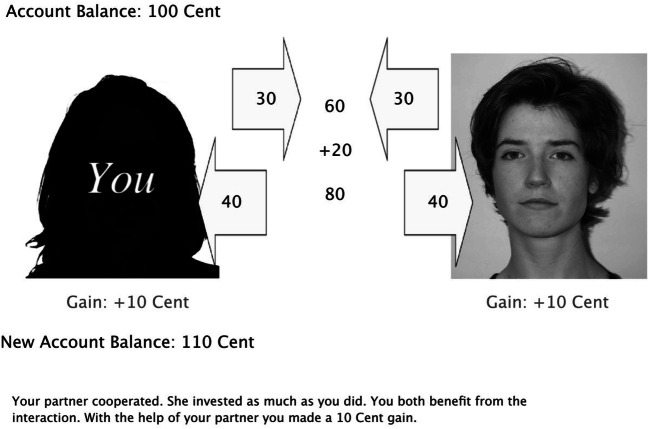
A screenshot of the sequential Prisoner’s Dilemma game. Here, both the participant and the partner cooperated and invested 30 cents which results in a 10 cents gain for each of them. The partner’s photograph shown in this example was taken from the Center for Vital Longevity (CVL) face database (Minear & Park, 2004)

A silhouette at the left side of the screen represented the participant (see Fig. 5). At the right side of the screen, a photograph of the partner was shown. In each trial, participants decided to invest either 15 or 30 cents by pressing a button on a response box. The decision was displayed on screen for 500 ms. For 500 ms, the investment was shown in an arrow which then moved to the center of the screen within an additional 500 ms. After 500 ms, the partner’s investment was shown in an arrow for 500 ms, which also moved to the center of the screen within 500 ms. After 500 ms, the sum of investments, a bonus of one-third of the sum and the total sum were presented in the center of the screen, each for 500 ms. After 500 ms, the total sum was split evenly between the partners, regardless of their previous investments. The partner’s share was shown in an arrow and moved toward the partner’s photograph (within 500 ms). After 500 ms, the participant’s share was shown in an arrow and moved toward the participant’s silhouette (within 500 ms). After 1 s, both interactants’ gains and losses, as well as the updated account balances (after 500 ms) were shown below the photograph and the silhouette, respectively. Participants gained money when interacting with cooperators, and lost money when interacting with cheaters. The losses that resulted from interactions with cheaters were as large as the gains that resulted from interactions with cooperative partners. A verbal description of the interaction was shown until the participant pressed a *continue* button on the response box to start the next trial. Two practice trials were provided to familiarize the participants with the game.

#### Judgments of item and source learning

Participants in the without-judgment group performed the prisoner’s dilemma game without providing metamemory judgments, as in previous studies (Bell et al., 2012; Mieth et al., 2016). Participants in the with-judgment group provided metamemory judgments immediately after each round of the prisoner’s dilemma game. Judgments were assessed as in Experiment 1. First, participants were asked to predict the probability that they would later remember the partner’s face (judgment of item learning). Second, they were asked to predict the independent probability that they would later remember the partner’s behavior (judgment of source learning).

#### Source monitoring test

After the game phase, participants received instructions for a standard source monitoring test (Bell et al., 2012; Mieth et al., 2016). The 40 faces from the prisoner’s dilemma game were randomly intermixed with 40 new faces from the same pool of faces. Twenty of the new faces were trustworthy looking and 20 were untrustworthy looking. All 80 faces were presented in random order, one at a time, at the center of the screen. For each face, participants first rated the likability of the face on a scale ranging from 1 (*not likable at all*) to 6 (*very likable*). Then participants were required to indicate whether they had seen the face in the game before. If so, they were asked whether the face was paired with cheating or cooperation. By pressing the *continue* button, participants initiated the next trial.

#### Measuring source memory

The multinomial source monitoring model (Bayen et al., 1996) was used to estimate parameters for item memory (*D*), item guessing (*b*), source memory (*d*), and source guessing (*g*). Here, Source A and Source B refer to cheating and cooperation in the prisoner’s dilemma game, respectively. Four sets of the model in Fig. 1 were needed for the combinations of faces (trustworthy looking and untrustworthy looking) and the two judgment groups (without judgments and with judgments).

#### Postdictions

After the memory test, participants provided postdictions (Schaper et al., 2019a) for all four combinations of facial trustworthiness (trustworthy looking and untrustworthy looking) and partner behavior (cheating and cooperation), as in Experiment 1.

#### Design

We used a 2 × 2 × 2 design with facial trustworthiness (trustworthy vs. untrustworthy) and partner behavior (cheating vs. cooperation) as within-subject factors. Judgment group (without judgments vs. with judgments) was again a between-subjects factor. Dependent variables were judgments of item and source learning averaged across items, objective memory (item and source memory) and postdictions of item and source memory.

### Results

Metamemory judgments and source monitoring processes were analyzed as in Experiment 1. Additionally, investments in the prisoner’s dilemma game and test-phase likeability ratings were analyzed with repeated-measures ANOVAs.

#### Investments in the prisoner’s dilemma game and likability ratings at test

Before focusing on metamemory and memory, it seems worth noting that both game investments and test-phase likability ratings sensitively reflected the manipulations of facial and behavioral trustworthiness, as expected (see Table 2). Participants invested more in the prisoner’s dilemma game when interacting with trustworthy-looking partners than when interacting with untrustworthy-looking partners, *F*(1, 183) = 142.84, *p* < .001, η_p_^2^= 0.44. At test, trustworthy-looking faces were rated as being more likable than untrustworthy-looking faces, *F*(1, 183) = 515.79, *p* < .001, η_p_ = 0.74, and cooperators were rated as more likable than cheaters, *F*(1, 183) = 56.84, *p* < .001, η_p_^2^= 0.24. Judgment group had no main effect on any of these variables and did not interact with any other factor (all *F*s ≤ 3.35). These findings suggest that facial trustworthiness and partner behavior were successfully manipulated, and cheating and cooperative behavior had a significant influence on the socioemotional evaluation of the partners.

**Table 2:** Means (and standard errors) for game investments and test-phase likability ratings as a function of the partner’s facial trustworthiness (trustworthy vs. untrustworthy), partner’s behavior (cheating vs. cooperation), and judgment group (without-judgments vs. with-judgments) in Experiment 2

	Without judgments		With judgments	
	Trustworthy	Untrustworthy	Trustworthy	Untrustworthy
	Game investments			
	22.13 (0.38)	18.42 (0.31)	21.73 (0.38)	18.67 (0.31)
	Likability ratings			
Cooperation	3.77 (0.07)	2.61 (0.06)	3.75 (0.07)	2.75 (0.06)
Cheating	3.59 (0.07)	2.46 (0.06)	3.51 (0.07)	2.44 (0.06)

#### Judgments of item and source learning

In the with-judgments group, judgments of item learning were neither influenced by facial trustworthiness, *F*(1, 91) = 1.01, *p* = .317, η_p_^2^= 0.01, nor by partner behavior, *F*(1, 91) = 1.27, *p* = .263, η_p_^2^= 0.01. There was no interaction between facial trustworthiness and partner behavior, *F*(1, 91) = 0.53, *p* = .467, η_p_^2^= 0.01 (see Fig. 2c).

For judgments of source learning, there were no main effects of facial trustworthiness, *F*(1, 91) = 2.04, *p* = .157, η_p_^2^= 0.02, and partner behavior, *F*(1, 91) = 1.16, *p* = .284, η_p_^2^= 0.01. However, critically, there was a significant interaction between facial trustworthiness and partner behavior, *F*(1, 91) = 25.69, *p* < .001, η_p_^2^= 0.22. On average, participants predicted better source memory for trustworthy-looking cooperators than for trustworthy-looking cheaters, *F*(1, 91) = 10.96, *p* = .001, η_p_^2^= 0.11, and for untrustworthy-looking cheaters than for untrustworthy-looking cooperators, *F*(1, 91) = 16.20, *p* < .001, η_p_^2^= 0.15 (see Fig. 2d). Thus, in their metamemory predictions participants expressed the belief that they would have better source memory for expected behaviors.

#### Item and source memory

We used the same equality restrictions for the base model as in Experiment 1. The base model fit the data well, *G*^2^(4) = 3.60, *p* = .462, which suggests that the restriction that item memory did not differ between cheater and cooperator faces (implied by the base model) is compatible with the data (cf. Barclay & Lalumière, 2006; Bell et al., 2012; Bell, Mieth, & Buchner, 2015; Mehl & Buchner, 2008; Mieth et al., 2016). However, item memory was higher for untrustworthy-looking faces than for trustworthy-looking faces, Δ*G*^2^(2) = 7.16 *p* = .028, *w* = 0.02 (see Fig. 3c). Furthermore, participants in the with-judgments group had better item memory than participants in the without-judgments group, Δ*G*^2^(2) = 57.17 *p* < .001, *w* = 0.06. This suggests that the metacognitive processing benefitted item memory, consistent with previous studies (Rhodes, 2016; Schaper et al., (2019a).

Source-memory parameter *d* represents the conditional probability that participants remember the cheating or the cooperation of a partner given that they have recognized the face as old. As in Experiment 1, we further restricted the model by using one parameter representing source memory for expected item–source pairings (trustworthy-looking cooperators and untrustworthy-looking cheaters) and one for unexpected item–source pairings (trustworthy-looking cheaters and untrustworthy-looking cooperators). The resulting model was compatible with the data, *G*^2^(8) = 5.81, *p* = .669. This new base model was used for the following analyses of the source memory parameters. Source memory for expected and unexpected source pairings differed significantly between groups, Δ*G*^2^(2) = 9.85, *p* = .007, *w* = 0.03. In the without-judgments group, participants showed an expectancy violation effect, replicating previous studies (Bell et al., 2012; Mieth et al., 2016; Suzuki & Suga, 2010). Source memory was better for unexpected than for expected item–source pairings, Δ*G*^2^(1) = 9.24, *p* = .002, *w* = 0.02 (left side of Fig. 3d). As in Experiment 1, this expectancy-violation advantage was not significant for participants who provided metamemory judgments at encoding, Δ*G*^2^(1) = 1.05, *p* = .306, *w* < 0.01 (right side of Fig. 3d).

Parameter *g*—which reflects the probability of guessing that a face was associated with cheating—differed significantly between trustworthy-looking faces and untrustworthy-looking faces, independent of whether participants provided metamemory judgments, Δ*G*^2^ (1) = 17.05, *p* < .001, *w* = 0.03, or not, Δ*G*^2^(1) = 47.12, *p* < .001, *w* = 0.06 (see Table 3). Participants in both groups guessed that trustworthy-looking partners had been associated with cooperation and that untrustworthy-looking partners had been associated with cheating.

**Table 3:** Guessing parameter estimates (and 95% confidence intervals) as a function of the partners’ facial trustworthiness (trustworthy vs. untrustworthy) and judgment group (without judgments vs. with judgments) in Experiment 2

Without judgments		With judgments	
Trustworthy	Untrustworthy	Trustworthy	Untrustworthy
Parameter *b*			
0.24 (0.21–0.26)	0.22 (0.19–0.24)	0.30 (0.27–0.33)	0.29 (0.26–0.32)
Parameter *g*			
0.38 (0.31–0.44)	0.71 (0.64–0.77)	0.45 (0.38–0.52)	0.66 (0.59–0.73)

*Note.* Parameter *b* reflecting the probability of guessing that a face was old. Parameter *g* representing the probability of guessing that the face of a partner was associated with cheating rather than cooperation

#### Postdictions of item and source memory

After the memory test, participants in both groups provided postdictions for all four cells of the design. Postdictions for item and source memory are shown in Figs. 4c–d. Participants thought that they had remembered trustworthy-looking faces better than untrustworthy-looking faces, *F*(1, 183) = 5.22, *p* = .024, η_p_^2^= 0.03. They also thought that item memory had been better for cheaters than for cooperators, *F*(1, 183) = 5.43, *p* = .021, η_p_^2^= 0.03. These main effects were qualified by a significant interaction, *F*(1, 183) = 6.46, *p* = .012, η_p_^2^= 0.03. Participants thought that item memory had been better for untrustworthy-looking cheaters than for untrustworthy-looking cooperators, *F*(1, 183) = 12.64, *p* < .001, η_p_^2^= 0.06, while no such difference was obtained for trustworthy-looking faces, *F*(1, 183) = 0.04, *p* = .835, η_p_^2^< 0.01. There was neither a main effect of judgment group nor any two-way or three-way interactions with this variable (all *F*s ≤ 0.20).

For the postdictions of source memory, there was no main effect of facial trustworthiness, *F*(1, 183) = 1.93, *p* = .167, η_p_^2^= 0.01. The main effect of partner behavior, in contrast, was significant—participants thought they had remembered cheating better than cooperative behavior, *F*(1, 183) = 18.36, *p* < .001, η_p_^2^= 0.09. The interaction was not significant, *F*(1, 183) = 3.60, *p* = .059, η_p_^2^= 0.02. There was neither a main effect of judgment group nor any two-way or three-way interactions with this variable (all *F*s ≤ 0.44).

### Discussion

Experiment 2 tested whether metamemory in source monitoring with socially relevant materials follows the same principles as metamemory for nonsocial materials. Most notably, Experiment 2 provided evidence of a metamemory expectancy illusion in social source memory. Participants on average predicted better source memory when the cheating or cooperative behavior of the partner confirmed their expectations about the trustworthy-looking or untrustworthy-looking person. In stark contrast, veridical source memory was better for unexpected behaviors than for expected behaviors in the without-judgment group. A similar metamemory expectancy illusion has been obtained in Experiment 1 and in other studies (Schaper et al., 2019a, 2019b) with nonsocial stimulus material. The present results demonstrate that people fall prey to the same metacognitive illusion even when making judgments about information that is socially relevant and associated with real financial gains and losses. This suggests that metamemory seems to be governed by similar principles for social and nonsocial information.

Another parallel to the findings of Experiment 1 and Schaper et al. (2019a) is that the source memory advantage for unexpected information was reduced when participants provided metamemory judgments after each encoding trial. This is also consistent with the finding of Soderstrom et al. (2015) that memory for related word pairs was selectively increased when participants provided judgments of learning. This finding thus strengthens the general conclusion that memory for cheaters and cooperators is determined by the same principles as memory for nonsocial information (Bell & Buchner, 2012).

Postdictions about item and source memory assessed immediately after test did not differ between the judgment groups in Experiment 2. In contrast to the in-the-moment judgments of source learning, source-memory postdictions were characterized by a main effect of partner behavior, suggesting that participants believed after test that they had remembered cheating better than cooperative behavior. The interaction between facial trustworthiness and partner behavior did not attain significance. This pattern of results is thus different from that obtained in Experiment 1. Other than the judgments of source learning obtained at encoding, the postdictions thus seem to differ between social and nonsocial stimuli. A potential explanation for this difference between postdictions and judgments of source learning obtained during encoding is that postdictions—as global judgments—are more reflective of the participants’ beliefs or naïve theories about memory, whereas judgments of source learning during encoding are more strongly determined by in-the-moment processing experiences (Frank & Kuhlmann, 2017). In fact, it seems plausible that people have different beliefs about social memory versus nonsocial memory. This explanation implies that people hold the belief that cheating is better remembered than cooperative behavior, which is plausible considering the salience of the negative experience of being cheated. However, this assumption needs to be tested. Experiment 3 therefore examines whether people hold the belief that cheaters are better remembered than cooperators.

## Experiment 3

Experiment 3 investigated beliefs about memory for socially relevant information. Global metamemory beliefs are often assessed by using vignette descriptions of previous experiments (e.g., Mueller, Dunlosky, Tauber, & Rhodes, 2014). In an online survey, we described the prisoner’s dilemma game used in Experiment 2 to the participants and asked them to express their beliefs about item and source memory without having experienced the game themselves.

### Method

#### Participants

The final sample was based on *N* = 100 students (86 female) of the University of Mannheim who participated in the online study. Age ranged between 18 and 37 years (*M*_*a*ge_ = 21, *SD*_*a*ge_ = 3). Seven additional participants did not complete the study and two additional data sets had to be removed because of repeated participation. Only fluent German speakers were allowed to participate.

#### Beliefs about item memory and source memory

First, participants read a description of the prisoner’s dilemma game that was used in Experiment 2. It was made clear that the partners in the game cooperated or cheated, and that they looked trustworthy or untrustworthy. Then the source monitoring test was described. After that, the participants in Experiment 3 were asked how well they believed the participants in the described study would remember the faces of their partners (item memory) and the behaviors of these partners (source memory). Separate judgments presented in randomized order were required for each cell of the 2 × 2 design of Experiment 2 in which facial trustworthiness (trustworthy vs. untrustworthy) and partner behavior (cheating vs. cooperation) were within-subject independent variables. For each of these four combinations, participants first provided their item-memory beliefs (e.g., “How likely was it that participants remembered the faces of trustworthy-looking people who cheated in the game? [Remembering here only means that participants remembered having seen the face; it does not matter whether they also remembered the behavior correctly.]”) and then their source-memory beliefs (e.g., “Staying with trustworthy looking cheaters: For what percentage of those persons whose face participants recognized did they correctly remember that they cheated in the game?”). Participants then proceeded to the next trustworthiness-behavior combination. As in Experiments 1 and 2, judgments ranged between 0% and 100%.

### Results

**Fig. 6:**
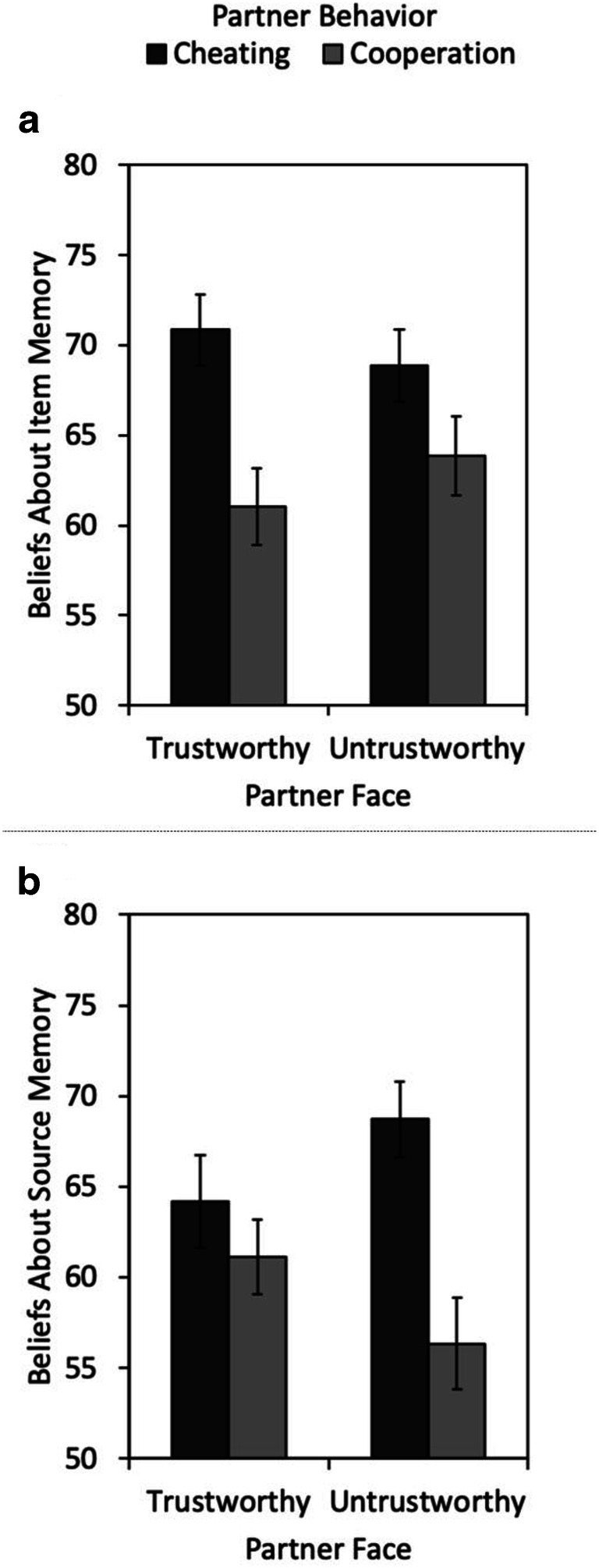
Global beliefs about item memory (**a**) and source memory (**b**) as a function of the facial trustworthiness (trustworthy vs. untrustworthy) and behavior (cheating vs. cooperation) in Experiment 3. The error bars represent the standard errors of the means

For item-memory beliefs, there was a main effect of partner behavior, *F*(1, 99) = 23.21, *p* < .001, η_p_^2^= 0.19. Participants thought that faces of cheaters would be better remembered than faces of cooperators. Neither the main effect of facial trustworthiness, *F*(1, 99) < 0.01, *p* = .928, η_p_^2^< 0.01, nor the interaction, *F*(1, 99) = 0.85, *p* = .359, η_p_^2^< 0.01, were significant (Fig. 6a).

For source-memory beliefs, the main effect of partner behavior was significant as well, *F*(1, 99) = 16.12, *p* < .001, η_p_^2^= 0.14. Participants thought that a partner’s cheating would be better remembered than a partner’s cooperation. There was no main effect of facial trustworthiness, *F*(1, 99) < 0.01, *p* = .942, η_p_^2^< 0.01. The interaction was not significant, *F*(1, 99) = 3.50, *p* = .064, η_p_^2^= 0.03 (see Fig. 6b).

### Discussion

As expected, source-memory beliefs obtained in Experiment 3 followed the same pattern as the source-memory postdictions for social stimuli obtained in Experiment 2. Most importantly, there was a significant main effect of partner behavior on the global beliefs about source memory, reflecting the belief that cheating is better remembered than cooperative behavior. These results therefore suggest that the source-memory postdictions in Experiment 2 could have been based on the participants’ beliefs. This is in line with the previous suggestion of Frank and Kuhlmann (2017) that postdictions, as aggregated judgments about memory, strongly reflect global beliefs about memory. By contrast, however, item-memory beliefs did not show the same pattern as the item-memory postdictions in Experiment 2. This may suggest that postdictions about item memory are not only based on global beliefs but are also, to some degree, modulated by experience-based factors. However, note that the evidence linking the global beliefs (assessed in Experiment 3) and the postdictions (assessed in Experiment 2) is only indirect as it involves a comparison across different experiments and samples.

## General discussion

The present experiments provide evidence for a metamemory illusion in people’s source-memory predictions for nonsocial and social stimuli. In Experiments 1 and 2, we presented information that either conformed or violated participants’ expectations. In Experiment 1, the to-be-remembered information was nonsocial in nature. Participants saw items that were either expected for a bathroom or a kitchen that were placed in a kitchen or a bathroom context. In Experiment 2, the to-be-remembered information was social in nature. Participants played a prisoner’s dilemma game with trustworthy-looking and untrustworthy-looking partners who either cheated or cooperated. In both experiments, participants provided metamemory judgments about item and source learning at encoding. Judgments of source learning consistently showed an expectancy illusion across experiments. Participants predicted better source memory for expected item–source pairings than for unexpected pairings. In Experiment 1, participants predicted to have better source memory for a typical kitchen item when it was placed in the kitchen rather than in the bathroom. Similarly, in Experiment 2, participants predicted better memory for the behavior of an untrustworthy-looking partner when the partner cheated than when the partner cooperated. By contrast, in both experiments veridical source memory was not in line with the expectations in the with-judgments groups, and it was even better for unexpected contexts in the without-judgment groups. The present findings thus replicate the novel finding of a metamemory expectancy illusion in judgments about source learning first reported by Schaper et al. (2019a). Extending these previous findings, we show for the first time that the metamemory expectancy illusion can also be found to the social domain.

The fact that the metamemory illusion generalized across experiments is particularly noteworthy when considering how different the nonsocial stimuli and social stimuli were from each other. Most noteworthy, the to-be-remembered cheating and cooperating behavior of the partners in Experiment 2 had real consequences for the participants in form of financial gains and losses, which was not the case when participants had to remember that, for example, an oven was presented in the bathroom in Experiment 1. A priori, it was unclear whether people would have accurate metacognitive awareness when the to-be-remembered information is socially and emotionally relevant. The present study, however, demonstrates that people have metacognitive misconceptions about their source memory even in situations with high (self-)relevance.

The similarities across experiments suggest that metamemory judgments about social and nonsocial information are partly governed by the same principles. Schaper et al. (2019a, 2019b) suggested that people rely on processing fluency as a mnemonic cue for making judgments of source learning. Specifically, information that is conceptually related and consistent with expectations is processed more fluently at encoding (Alter & Oppenheimer, 2009; Undorf & Erdfelder, 2011, 2013, 2015). Experiencing fluency at encoding leads people to predict better memory performance. Ironically, unexpected information is often processed more thoroughly precisely because it violates one’s established schematic and stereotypic expectations, which leads to higher veridical memory for unexpected relative to expected source information (Graesser & Nakamura, 1982; Küppers & Bayen, 2014). These mechanisms may be identical for social and nonsocial information. This assumption is parsimonious as it seems unnecessary to invoke different mechanisms to explain the same pattern of findings across domains. From an evolutionary point of view, it can be argued that relying on domain-general cognitive machinery for problems whose structure is identical across different domains may be more efficient than using a plethora of separate but functionally identical modules (Bell & Buchner, 2012). However, based on the present results, it cannot be completely ruled out that the underlying processes differ across domains but have certain principles in common.

The finding of a metamemory illusion in judgments about adaptively relevant material raises the question of whether or not metacognitive illusions are linked to maladaptive cognition and behavior. When it comes to metacognition, it has long been recognized that introspective awareness of the rules governing complex cognitive processes is not always necessary to achieve satisfactory levels of performance. For example, a baseball player may be perfectly able to catch a ball without being able to reproduce the complex algorithms that are necessary to predict where the ball will hit the ground (Todd & Gigerenzer, 2012). A lack of metacognitive awareness thus does not necessarily represent evidence of a maladapted system. Relying on schema-based guessing leads to a high number of source errors in experiments in which a high proportion of schematically unexpected information is presented, but these artificially determined contingencies may not be representative of natural environments. In principle, the simple strategy of using prior knowledge to get at source attributions could be highly efficient in those environments from which the schematic expectations have been derived (Johnson et al., 1993). The metamemory illusion in the judgments of source learning identified by Schaper and colleagues (2019a)—that was replicated here—can be explained by assuming that people have no awareness of the relative contributions of memory and guessing to their source attributions. Pinpointing the exact circumstances under which this lack of metacognitive awareness produces adaptive or maladaptive consequences is an interesting avenue for future research.

Another parallel between Experiments 1 and 2 is the reactivity of source memory to metamemory judgments. Source memory was influenced by the provision of metamemory judgments in the encoding phase. Memory for schematically expected source information in particular benefitted from the metamemory judgments, resulting in an attenuated expectancy violation effect in veridical source memory. This finding fits well with the observation that the requirement of having to provide metamemory judgments selectively increases memory for related information in word-pair learning (Soderstrom et al., 2015). A possible explanation for this finding is that relatedness is used as a cue for judgments of source learning which may cause attention to be drawn to the consistency between the items and the sources. The requirement to give metamemory judgments may therefore strengthen memory for expected information. In line with this explanation, Schaper et al. (2019a) found that the attenuation of the expectancy-violation effect could be traced back to providing judgments of source learning, but not to providing judgments of item learning. Thus, in our experiments, a comparatively subtle manipulation oriented attention to expected information and diminished the source memory advantage for unexpected item–source pairings. Thereby, our results further corroborate that a memory advantage for trustworthy-looking cheaters is most likely *not* caused by a highly specialized cheater-detection module (Suzuki, Honma, & Suga, 2013; Suzuki & Suga, 2010) but is rather based on flexible memory-and-attention mechanisms (Bell & Buchner, 2012; Bell et al., 2015). Specifically, due to the salience of unexpected information, people may allocate more attention to unexpected item–source pairings, but this bias is eliminated when metamemory instructions direct attention in the opposite direction.

Even though most of the results of Experiment 1 were replicated in Experiment 2, there were also some noticeable differences. First, an expectancy illusion in the judgments of source learning was obtained in both experiments, but it was less pronounced in Experiment 2 than in Experiment 1. These differences in the absolute size of the effect are difficult to interpret due to the methodological differences between the experiments, but they suggest that the factors underlying the expectancy illusion may differ in strength between the experiments. Secondly, only judgments of source learning showed an expectancy illusion in both experiments while the judgments of item learning differed between experiments. Specifically, there was an expectancy illusion in the judgments of item learning in Experiment 1, but this pattern was not replicated in Experiment 2. This strengthens the conclusion of Schaper et al. (2019a) that judgments of item learning differ from judgments of source learning. The finding suggests that the metamemory judgments of item learning are less affected by the encoding context when they refer to faces than when they refer to words. Despite this obvious difference in the pattern of results between the experiments, it seems noticeable that the expectancy illusion was stronger in the judgments of source learning than in the judgments of item learning in Experiment 1 as well. When analyzing the judgments of learning in a 2 × 2 × 2 analysis with item types (bathroom items vs. kitchen items), source type (in the kitchen vs. in the bathroom) and judgment type (judgement of item learning vs. judgment of source learning) as within-subjects variables, a significant three-way interaction was revealed, *F*(1, 59) = 45.96, *p* < .001, η_p_^2^= 0.44. This result confirms the finding of Schaper et al. (2019a) that the expectancy illusion is generally stronger in the judgments of source learning than in the judgments of item learning.

Another interesting difference between the results of the first two experiments is that the postdictions differed across experiments. A potential explanation for this pattern of finding is that postdictions, as global judgments, are more strongly affected by the participants’ beliefs. There is little reason to assume that people hold the same beliefs about the factors that determine their social and nonsocial memory. Indeed, Experiment 3 shows that people hold the belief that cheaters are particularly memorable. This is different to the nonsocial domain in which participants expressed the belief that expected information is better remembered than unexpected information (Schaper et al., 2019b). To the degree that metacognitive judgments are affected by these beliefs, differences between judgments about social information and judgments about nonsocial information are to be expected. Interestingly, the judgments of source learning obtained at encoding in Experiment 2 were characterized by an expectancy illusion, with no main effect of partner behavior. The judgments of learning thus did not reflect the global belief that cheating is remembered better than cooperation. This pattern of results may suggest that the judgments of source learning were not strongly determined by participants’ global beliefs, but rather—at least to some degree—by the participants’ in-the-moment experiences in the prisoner’s dilemma game.

In sum, the present study replicates and extends previous studies on metamemory (Schaper et al., 2019a; Undorf & Erdfelder, 2015) by examining metamemory judgments for nonsocial information (expected or unexpected items in the kitchen and the bathroom) and socially relevant information (other people’s faces and behaviors). We found that people are prone to a metamemory illusion in source memory in that they tend to predict enhanced source memory for bathroom and kitchen items in expected rooms (Experiment 1) and social behaviors that are consistent with stereotypic expectations (Experiment 2). Further, without encountering social partners, people seem to hold the general belief that cheating is better remembered than cooperation (Experiment 3). In contrast to these metamemory judgments, objective source memory is usually enhanced for unexpected information (Bell & Buchner, 2012). This replicates the novel finding of a metamemory illusion in judgments of source learning (Schaper et al., 2019a). Extending previous research, the present study shows that this metamemory illusion generalizes to the social domain. Not only is memory for cheaters determined by general principles (Bell & Buchner, 2012; Bell et al., 2015), the present results suggest that the same conclusion does—at least to some degree—apply to metamemory as well.

### Open practices statement

The data of the experiments are available at https://osf.io/h98qs/

## Footnotes

1 We also performed supplementary hierarchical Bayesian analyses for Experiments 1 and 2 with TreeBUGS (Heck et al., 2018) that take individual variability into account, which confirmed the results based on the aggregated data.
